# Bibliographical Notices

**Published:** 1844-05-04

**Authors:** 


### BIBLIOGRAPHICAL NOTICES.

*A Treatise on Operative Surgery; comprising a description of the various processes of the art, including all the new operations, exhibiting the state of surgical science in its present advanced condition; with eighty plates, containing four hundred and eighty-six illustrations.* By Joseph Pancoast, M. D., Professor of General, Descriptive, and Surgical Anatomy, in Jefferson Medical College, Philadelphia; Lecturer on Clinical Surgery; one of the attending Surgeons at the Philadelphia Hospital, &c., &c. Philadelphia, Carey and Hart, 1844, large quarto, pp 380.

We have just received from the publishers, a copy of this elaborate work. It is altogether one of the most splendid and expensive publications that have issued from the American press. The operations in Surgery, from the simplest to the most difficult and terrible, are shown with a care and precision, and in a manner so clear and demonstrative, that it would seem as if the verriest tyro might perform them after an inspection of these illustrations. The various modes of operating practised by different Surgeons in the several cases are exhibited, so as to afford to the practitioner an opportunity of choosing the plan which may accord best with his own views. In order to render the work still more useful to the practitioner, a brief but comprehensive description of the Surgical Anatomy of the parts immediately concerned has been added, as well, also, as some account of the pathological changes, when this was deemed neccessary to the comprehension of the operation in question. For the accuracy of the descriptions, Anatomical and Surgical, the profession need no higher guaranty than the well known character of the author as an experienced Surgeon and profound Anatomist.

The mechanical execution of this important work, does honor to American Artists. The figures are drawn with great beauty and fidelity by some of the first artists, and lithographed by Duval, in his best manner. The text is printed in double column, on good white paper, with new and handsome type. Altogether, it is a splendid production of American enterprise and talent.

---

*A Practical Treatise on Midwifery.* By M. Chailly, Doctor of Medicine, and ex-chief of the Obstetrical Clinique of the Faculty of Paris, Professor of Midwifery, Member of the Society of Medical Emulation, &c. *Illustrated with* 216 *wood cuts. Translated from the French and Edited by* Gunning S. Bedford, A. M., M. D., Professor of Midwifery and the Diseases of Women and Children, in the University of New York. 8vo. pp 630. New York, Harper & Brothers, 1844.

[illegible] great fault of the generality of French publications is t[illegible] ignorance or neglect they exhibit of every thing tha[illegible]s not *French*. Paris is not only all France, but all the [illegible]vorld. The exceptions to this remark among their late [illegible]riters on medicine are too few, and the instances of d[illegible]arture too rare, to admit of much cavil on the subject. The present volume affords a striking example in this [illegible]spect. Among the British authorities, as far as we h[illegible]e observed in a rapid perusal, Montgomery is cited once; of those on the continent, Nægele and Stoltz, perhaps once or twice, and that is about all out of France. American authorities are altogether overlooked. And yet this work is "offered to the student, as combining all that is new and valuable in obstetric science!"

Chailly ("Honoré,") is a young obstetrician of Paris, until the appearance of this work, quite unknown to fame. He represents himself as a protégé of Paul Dubois, and the present treatise is an exposition of the views of that Professor, chiefly, and, as we have said, with but little reference to those of any body else, except, indeed, Dubois, the older, and Honoré, the father-in-law of Chailly. It is very well known to all who are well read in obstetrical science, that since the time of Baudelocque, the French have contributed little of real importance to our knowledge in this department of the profession, and M. Paul Dubois, eloquent, accomplished and popular as he may be as a lecturer, derives much of his reputation from his father, and the official position which he occupies. Can any one point to a great principle, or important fact that he has added to our knowledge of obstetrics?

It was to be expected of an American Editor, when preparing a work for American physicians, that he would have supplied the obvious defects of one so defficient; that he would have enriched it from the abundant stores afforded by British and German publications, and that he would, at least, have Americanized it in some degree. The Editor, it appears thought differently. He has, therefore, not made such alterations or additions. It is true, his notes are not very extensive, and he has given us his own experience pretty largely in what he has added, which is of course very important. But was it unreasonable to expect some notice of the writings of such men as Denman, Blundell, Rigby, and Churchill, among the British? or, if such "outside barbarians" as James, Dewees, and Meigs, are undeserving of such attention, might we not with some reason look for the opinions and experience of Bard, of Francis, of Delafield, of Gilman, among the distinguished, in the "Commercial Metropolis?"

We have not observed the names of the latter in the book, and the former are referred to only, we believe, in connexion with a remarkable case of Cæsarean section. However, after the "friendly warning," ("not by way of threat") which has been kindly extended to us of the "Medical Metropolis," it may be "safe no longer" to scan too closely what comes from "certain quarters," and therefore that we may not bring ourselves "under condemnation," we shall abstain from further complaint, and hasten to present to our readers some of the Editor's notes, as examples of his matter and style.

*Active motion of the Fœtus.*—Women who have never borne children, and whose desire has been to have offspring, are generally very apt to imagine themselves pregnant; and there are few symptoms about which they are more readily deceived than the active motion of the fœtus. The accoucheur should never rely upon any statements made by his patients in cases of doubtful pregnancy, especially if any important interest, such as character or property, be involved in the issue. It is his duty to judge for himself, irrespective of all adventitious or other influences; let his mind be free from all bias, and judge of the case according to the evidence which may be presented to his senses. Such is the rule of conduct I would most earnestly enjoin on all who may wish to discharge their trust fearlessly, and, at the same time, justly. A most amusing case occurred in this city some ten years since, and will, perhaps, serve more effectually to illustrate an important truth in midwifery than any argument I could advance. A lady, aged forty-seven, who had been married since her thirtieth year, had entertained a most anxious desire to become a mother, but had not succeeded in her wishes, and was about abandoning all hope, when, of a sudden, she noticed that her abdomen began gradually to enlarge, and she really imagined herself pregnant. In addition to the ordinary symptoms of gestation, she thought she distinctly felt the motion of her child. She received the congratulations of her friends, was complimented on her prowess and the final accomplishment of her hopes after years of fruitless effort, and commenced making the necessary preparations for her approaching accouchment. Her physician was sent for, and was informed that his services would be required, &c. In the course of a few months the labour commenced; a messenger hastened to admonish the doctor that the lady's time had come, with a request that he would lose no time in reaching the bedside of his delighted patient. The doctor arrived—all in the house was confusion—the nurse was enchanted—the husband could scarcely realize the advent of this long expected era in his life—the patient was in actual labor—the pains frequent and distressing—the physician was entreated to lose no time in assisting madam—he instituted an examination—the silence of death now pervaded the lying-in chamber to hear from the lips of the oracle the exact facts of the case. They were soon made joyful by hearing from the doctor that all was right—that the labor was quite advanced, and in a very short time would be completed. The sufferings of the patient increased—she was requested to make the most of her pains—to *bear down and assist nature*—when lo! in the midst of one of those powerful efforts to "assist nature," there was heard an explosion, which struck terror into the hearts of all present, the doctor included. The patient immediately exclaimed, "*Oh! dear doctor, it's all over; do tell me if it is a boy.*" The explosion was nothing more than an escape of air from the bowels, the patient having mistaken flatulence for pregnancy, and the rumbling of the gas in the intestines for the motions of the fœtus.

In 1839 I received a note, requesting me to visit, without delay, a lady who was residing in the State of New Jersey, about thirty miles from this city. I immediately repaired to her residence, and on my arrival was received by her father, a venerable and accomplished gentleman. He seemed broken in spirit, and it was evident that grief had taken a deep hold of his frame. On being introduced into his daughter's room, my sympathies were at once awakened on beholding the wreck of beauty which was presented to my view. She was evidently laboring under phthisis, and it appeared from her wasted form that her days were numbered. My presence did not seem to produce the slightest disturbance, and she greeted me with this expression, "Well, doctor, I am glad to see you on my beloved father's account, for he will not believe that I cannot yet be restored to health. Life has lost all its charms for me, and I long for the repose of the grave." These words were spoken with extraordinary gentleness, but yet with a firmness that at once give me an insight into the character of this lovely woman. From her own lips I received the following history of her case. Her father was a clergyman of high standing in the English Church, and had had a pastoral charge in England, in which he continued until circumstances rendered it necessary for him to leave that country aud seek a residence in America. At a very early age she had lost her mother, and was almost entirely educated hy her father, whose talents and attainments admirably fitted him for this duty. When she had attained her eighteenth year, there was an attachment formed between her and a young barrister of great promise and respectability. This attachment resulted in a matrimonial engagement. Soon after the engagement, she began unaccountably to decline in health. There was considerable irregularity in her menstrual periods, with more or less constant nausea, loss of appetite, inability to sleep, feverishness, and an uncontrollable dislike to society; besides, there was a general change in her personal appearance: her abdomen became enlarged, her breasts increased in size, &c. These changes soon attracted the attention of some of her female acquaintances, and rumour was uncharitable enough to suspect her virtue. The barrister to whom she was affianced heard of these reports, and, without a moment's delay, wrote a letter to her father, begging to be exonerated from his engagement. This was assented to without hesitation. This young lady, conscious of her own innocence, requested that a physician should be sent for, in order that the nature of her case might be ascertained. A medical man accordingly visited her, and, after an investigation of her symptoms, he, no doubt from very philanthropic motives, informed the father that she was pregnant, and that means should be immediately taken to keep the unpleasant matter secret. The father, indignant at this imputation against the honour of his child, spurned the proposition, and instantly requested an additional consultation. This resulted in a confirmation of the opinion previously expressed, and the feelings of that father can be better appreciated than described. Without any delay that good man determined to resign his living, gather up his little property, and proceed with his daughter to America. On his passage to this country his daughter became extremely ill, and as there was a physician on board the steamer, his advice was requested. After seeing the patient (she was at the time laboring under excessive vomiting from sea-sickness,) he told the father that he apprehended that she might have a premature delivery. Such, therefore, was the general appearance of this lady, that a physician, merely judging from *appearances*, at once concluded she was pregnant.

This was about the substance of what I learned of the previous history of this lady; and my opinion was then requested as to the character of her malady My feelings were naturally very much enlisted in her behalf, and I proceeded with great caution in th[illegible] investigation of her case.

In placing my hand upon the hypogastric regio[illegible], previously flexing the thighs upon the pelvis, I d[illegible]tinctly felt an enlargement, which, from its circu[illegible]scribed character and position, I recognised to be [illegible]e uterus. The abdomen was excessively tympani[illegible], and as much distended as it usually is at the sev[illegible]h month of gestation. In making a vaginal exam[illegible]a-tion, the enlarged uterus was plainly felt, and it [illegible]e-sented a hardness to the touch which I had n[illegible]er before experienced. It was not the hardne[illegible] of *schirrus*, nor was there any pain experience[illegible] on pressure. The os tincæ was slightly dilate[illegible]nd perfectly circular. Projecting through it I [illegible]t a small body, which to the touch seemed to b[illegible]f a calcareous nature. I could readily grasp the [illegible]rus between one hand applied to the hypogastric region and the finger introduced into the vagina; and it appeared to equal in size the fœtal head at full term. The organ appeared to be much more rounded in its development than is usual in pregnancy; and on introducing the finger into the rectum, the same hardness of the tumour was experienced that was observed in the vaginal examination, but an entire freedom from pain. It was very evident that there was no pregnancy, and without hesitation I gave this as my opinion; at the same time stating that the enlargement of the womb depended most probably upon some internal growth, the precise nature of which I could not determine. That it was not a polypus was evident from the absence of all the symptoms which usually characterise it. After I had expressed my opinion that she was not pregnant, the only observation this interesting creature made was, "Doctor, you are right." These few words were full of meaning, and their import I could not but appreciate. The father was soon made acquainted with the result of my examination, and he seemed perfectly unmoved as I expressed to him my opinion. It was evident that he had never faltered for one moment in the belief of his daughter's virtue, and required no assurance from me or any other living being that his child had been cruelly abused. He asked me with great solicitude whether something could not be done to restore her to health; and his agitation was extreme when I told him that his daughter was in the last stage of consumption. I left him with the pledge that he would inform me of her dissolution, and afford me an opportunity, by a post-mortem examination, of testing the truth of my opinion. About four weeks from this time I received a note announcing the death of this lady, and requesting that I would immediately hasten to the house, for the purpose of making the autopsy. Dr. Ostrom, now practising in Goshen, in this state, at my request accompanied me, and assisted in the examination. It was discovered that the enlargement of the uterus was in consequence of a fibrous tumour, which occupied its whole internal surface; and the small body which I had felt projecting through the os tincæ was a calcareous concretion, of which there were several attached to the tumour.

We cannot afford space at present for further quotations. These, probably, will suffice to gratify the curiosity of such of our readers as may not have the good fortune to see the work,—of which we can only say, that, like other treatises on the same subject, ancient and modern, it of course contains much of which we can approve, but nothing of the kind, so far as we have discovered, that is peculiar either to the author or his translator; whilst there are some things in it which we regard as decidedly objectionable.

In mechanical execution, the present publication is not likely to add much to the reputation of the American press. The illustrations, originally only *outlines*, are coarse wood engravings, very inferior to what we are accustomed to see of late in English and American publications on the same subject.

---

*Cyclopædia of Practical Medicine*, *Part II*. Apoplexy (Cerebral)—Bathing.

Messrs. Lea and Blanchard, with their accustomed punctuality, have already issued the second part of this valuable work, of which we gave a brief notice, in the last number of the Examiner.

This *part* consists of about 130 pages, containing thirteen articles, by various writers. Some of the articles are written with great ability, and almost any one that might be selected is well worth the cost of the whole.

---

*State of the New York Hospital and Bloomingdale Asylum, for the year* 1843.

This is the annual report of the "Governors," or board of managers by whom these institutions are controlled, and like all documents of the kind, it contains much to interest the physician and philanthropist. *The Hospital* is situated in the city of New York, and is devoted to the relief of medical and surgical patients. The medical administration is confided to a number of consulting, attending, and resident physicians and surgeons, among whom we recognize some of the most distinguished members of the profession in that city.

"The number of patients in this Institution on the 31st of December, 1842, was one hundred and ninety-eight, and there were admitted during the year 1843, one thousand one hundred and two, making a total of two thousand one hundred persons, who have received the benefits of the Hospital during the year."

*The Bloomingdale or Lunatic Asylum* is located near the East river, a little way out of the city-proper. The number of patients in this Institution on the 31st of December, 1842, was one hundred and ten, and eighty-five were admitted during the succeeding year; making a total of one hundred and ninety-five persons who have been under treatment in the Asylum during 1843.

"This Institution is under the immediate care of a resident Physician, a Warden, and a Matron. The general supervision is confided to a Committee of the Governors, part of whom visit the establishment at least weekly, and the whole Committee once in each month, when a report is made to the Board. Monthly visits are also made by the President, Vice President, and Inspecting Committee of the Hospital, who, in like manner, report to the Board of Governors.

"The unfortunate class of persons sent to this Institution are treated with all the tenderness which is compatible with their personal safety; and the medical and moral remedies, which long experience have indicated, are steadily, and in numerous instances, successfully applied to restore them to rational society. But, adds the report, it is still cause of deep regret that, in too many cases, the mistaken kindness or delicacy of friends, prevents that early application of restorative means, which is so essential to a favourable result."

Dr. Pliny Earle, formerly resident Physician at Friends Asylum, near Frankford, in the county of Philadelphia, has recently been appointed Rresident Physician to this Institution. Dr. Earle has had much experience in the treatment of the insane, and is otherwise well qualified for the discharge of the duties of the station to which he has been appointed.

---

DEAFNESS OF TUBERCULOUS SUBJECTS.

Impaired power of hearing, or actual deafness, is occasionally observed in tuberculous subjects, and is traceable, according to M. Ménières, to the development of tubercle in the substance of the membrane of the tympanum, leading to its more or less complete destruction.—*Lon. Med. Times.*

